# Recent advances in bioactive hydrogel microspheres: Material engineering strategies and biomedical prospects

**DOI:** 10.1016/j.mtbio.2025.101614

**Published:** 2025-02-25

**Authors:** Junjiang Yue, Zhengbiao Liu, Lu Wang, Miao Wang, Guoqing Pan

**Affiliations:** a Institute for Advanced Materials, School of Materials Science and Engineering, Jiangsu University, 301 Xuefu Road, Zhenjiang, Jiangsu 212013, China; b Department of Orthopedics, Suzhou Industrial Park Xinghu Hospital, No. 1 Tingsheng Street, Suzhou, Jiangsu 215000, China

**Keywords:** Hydrogel microspheres, Microfluidics, Biofunctionalization, 3D cell culture, Biosensing, Tissue regeneration

## Abstract

Hydrogel microspheres are a class of hydrophilic polymeric particles in microscale, which has been developed as a new type of functional biomaterials for wide-range biomedical applications in recent years. This review provides a comprehensive overview of the preparation methods for hydrogel microspheres, including droplet microfluidics, electrospray and emulsion was first summarized. At the same time, we analyze the impacts of these methods on the properties of hydrogel microspheres and explore various functionalization strategies for enhancing their bioactivity and expanding their biomedical applications. In addition, we discuss the recent advances and the further prospect of hydrogel microspheres in life science applications, particularly in cell biology research, bioanalysis and detection, as well as tissue repair and regeneration. By synthesizing the latest developments, this review aims to offer valuable insights and strategies for optimizing hydrogel microspheres in diverse application scenarios and inspire future research and practical innovations.

## Graphical abstract

**Figure:**
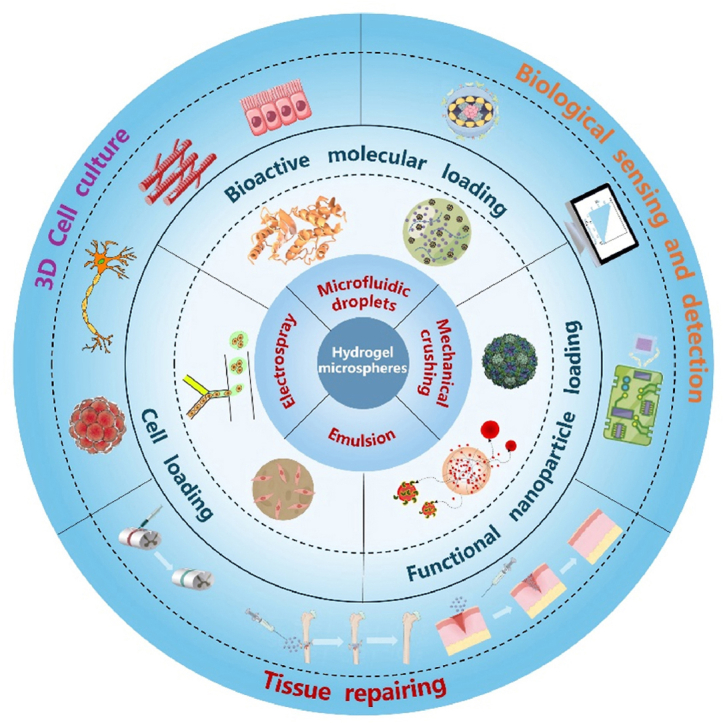

## 1 Introduction

Hydrogels are a class of three-dimensional network-structured hydrophilic polymers which are formed through a variety of crosslinking methods. The high water content, biomechanical adaptability, high permeability, controllable biodegradability makes hydrogel materials great promise for applications in biomedical science [[1], [2], [3], [4], [5]]. However, commonly bulk hydrogels are difficult to apply to numerous and complicated medical scenes with special requirements, due to their weak adaptability, low mechanical strength, limited transmission mode, especially the swelling and degradable problems with uncertain size changes in vivo [6,7]. Thus, the potential of hydrogel to adapt to diverse biomedical scenarios is severely limited. In this context, miniaturized format of hydrogels like hydrogel microspheres were derived. The high-water content of hydrogel microspheres enables their mechanical properties to closely mimic those of natural tissues, such as cartilage or skin, thereby reducing mechanical stress after implantation and promoting cell adhesion and proliferation. For example, hyaluronic acid hydrogel microspheres have been effectively utilized in joint cavity injections to improve the lubrication environment in osteoarthritis. Moreover, the surface of microspheres, whether composed of natural materials (e.g., chitosan, sodium alginate) or synthetic polymers (e.g., polyethylene glycol, PEG), can be modified to enhance their functionality. Surface modifications such as grafting with RGD peptides can promote cell-specific recognition and minimize the risk of inflammation, further improving the biocompatibility and therapeutic efficacy of hydrogel microspheres [8,9]. Due to the micro- and even nano-scale, hydrogel microspheres exhibit unique characterizations and have been widely used in various biomedical fields. As a type of formatted hydrogels, the hydrogel microspheres have shown promise not only as carriers or scaffolds for drug delivery and tissue repair, but also as the 3-dimensional (3D) cell culture supports by simulating cell growth microenvironment in vivo [[10], [11], [12], [13]]. Therefore, current studies on the preparation, functionalization, and applications of hydrogel microspheres have garnered significant attention across various fields, including physics, chemistry, materials science, biology, and medicine [14,15].

Up to now, the construction of hydrogel microspheres has been extensively explored. Various material engineering strategies from the screening of raw materials, functional modification to the preparation processes have been compared and optimized. Precursor materials for preparing hydrogel microsphere commonly use biocompatible or biogenic polymers, including gelatin, hyaluronic acid, polyethylene glycol, alginate, agarose and even the extracellular matrix (ECM) [[16], [17], [18], [19], [20]]. In addition to the bio-applicability, these polymeric materials are also convenient for late-stage biomodification due the abundant active groups (e.g., the hydroxyl, amido, sulfhydryl groups). Currently, there are several typical technologies, including droplet microfluidic techniques, electrospray and emulsion methods, having been developed for fabricating microscale spherical hydrogels. With the development of these micro-fabrication technology, hydrogel microspheres from a few microns to hundreds of microns could be easily obtained. It is important to note that particle size plays a critical role in the retention and clearance of microspheres in target tissues. For instance, microspheres smaller than 10 μm are more readily phagocytosed by macrophages, which may result in faster clearance. Conversely, microspheres larger than 200 μm are more likely to induce fibrous encapsulation, potentially limiting their effectiveness and prolonging their presence in the tissue. These size-dependent behaviors underscore the need for careful optimization of particle size for specific therapeutic applications. The principles and characterizations of all these methods are very imaginative, subtle and flexible, these ingenuities thus exhibit high adaptability to diverse requirements in biomedical fields [[21], [22], [23]].

As mentioned above, micro-scale materials may show some structure and scale-related unique properties. When hydrogel microspheres are used as drug carriers, the intrinsic characteristics will facilitate a sustained drug release and also the convenience for surface modification using targeting molecules. These advantages may lead to not only long-term biological effects but also well-designed cell or tissue specificity in practical uses. In addition, the miniaturized format of hydrogel microspheres also facilitates minimally invasive implantation using an injection syringe. Especially for tissue repair, the microscale spherical hydrogels can be easily injected to fill the defect of a wound site with high geometric adaptability. Therefore, the studies on surface biomolecular decoration, the sizes and internal porous properties of hydrogel microspheres are of great significance. The final results of these parameter optimizations will lead to an improved mass transfer rate in biological detection with enhanced analysis efficiency, or a high adaptability to different cell growth microenvironment improved tissue repair and regeneration. Currently, studies on the above parameters occupy the focus of most research, while a comprehensive summary is not yet available. Therefore, in addition to the control of size and porousness, the modification of hydrogel microspheres using different bioactive sources including biomolecules, functional nanomaterials, and even cells will also very important.

**Scheme 1:**
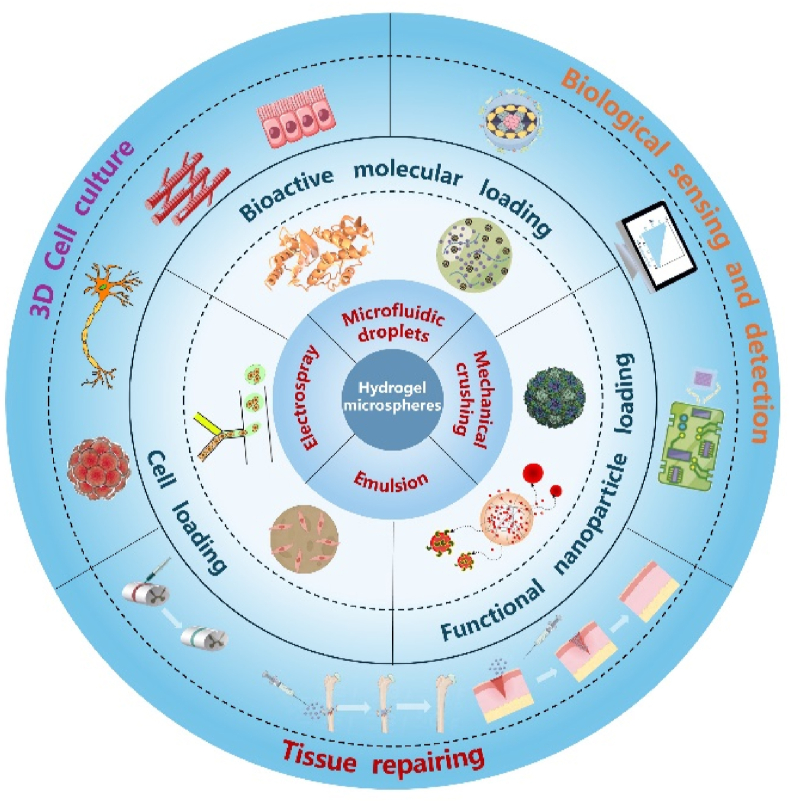
The preparations, biofunctionalizations and applications of hydrogel microspheres.

The biomedical potentials of hydrogel microspheres have been continuously expanded in recent years, due to the innovation of preparation technology and the enrichment of chemical means. It has been demonstrated that functional hydrogel microspheres have shown great promise in biological detection and sensing, cancer therapy, organ repair and tissue regeneration, etc. In addition, as cell scaffolds, the independent unit and miniaturized porous structure of a single microsphere may also endow the multi-functional hydrogel microspheres with ECM-mimicking 3D microenvironment, unique advantages in cell biology research. These potential applications clearly indicated the fabrication of bio-functional hydrogel microspheres is of great significance. In this review, we provide a general overview of recent advances in bioactive hydrogel microspheres. The preparation process and method of hydrogel microspheres, the general means for functionalization, and the typical examples of biomedical application in different scenarios are covered (Scheme 1). It is hoped that this review paper can inspire subsequent researchers to develop more material engineering strategies, and continually expand the prospects of hydrogel microspheres in life sciences in future.

## 2 Material engineering strategies for preparation of hydrogel microspheres

The physicochemical properties and biological effects of microspheres hydrogel are closely related to the diameter of microspheres in addition to the composition. Thus, the preparation methods of hydrogel microspheres are quite important. To obtain hydrogel microspheres with suitable and comparable size, a variety of material engineering strategies, including droplet microfluidic technology, emulsion method, electrospray method and mechanical stirring method were used [24,25]. The details of the engineering mechanism and the preparation process can be seen in below. In addition to some straightforward methods such as mechanical stirring-triggered phase separation, the microfluidic chip-related methods capable of steady determination of the microsphere size will be also discussed. In all these strategies, the key elements in the preparing process of hydrogel microspheres, especially the core components for different microfluidic droplet technology will be analyzed and discussed in detail.

### 2.1 Microfluidic droplet technology

Microfluidic droplet technology is not the first developed but the most widely employed method for preparing hydrogel microspheres, through the precise design of microchannels to control fluid dynamics, have achieved accurate regulation of the size and structure of hydrogel microparticles. In recent years, significant breakthroughs have been made in scaling up production capacity, making microfluidic methods the mainstream technology for the preparation of high-performance microparticles in the biomedical field [26]. The fundamental principle underlying microfluidic droplet technology is the utilizing of the shear force between different phases (e.g., water phase or oil phase, W/O) to disperse the solution into dispersed droplets. Hydrogel microspheres thus can form after polymerization when the water phase channel is flowed with is cross-linkable hydrophilic polymer solution. According to this principle, it is possible to produce tiny droplets with simple water-in-oil (W/O) structures, as well as hydrogel microbeads with more complex structures like oil-in-water-in-oil (O/W/O) [27]. One advantage of this method is the size controllability of hydrogel microspheres [28]. The regulation of several key parameters in the preparation process, such as phase flow rate, chip design, and the choice of hydrogel and crosslinking method, will all bring great effects on the structure, morphology and physical-chemical characteristics of the microspheres. Microfluidic technology offers a highly efficient and versatile approach for the fabrication of hydrogel microspheres, enabling precise control over their size, shape, and internal architecture with exceptional reproducibility. Its high-throughput capabilities allow for the rapid production of monodisperse microspheres, overcoming the inconsistencies associated with conventional fabrication methods. Furthermore, microfluidics provides a unique platform for in situ functionalization, facilitating the incorporation of bioactive molecules, responsive elements, and targeted ligands during the microsphere formation process. This not only enhances the stability and bioactivity of hydrogel microspheres but also enables stimuli-responsive drug release, intelligent therapeutic responses, and precise targeting for regenerative medicine and drug delivery applications. By integrating microfluidic techniques with advanced biomaterial design, hydrogel microspheres can be tailored for next-generation biomedical applications, including precision medicine and adaptive tissue engineering.

Thus, the resultant microsphere materials has been widely applied in many fields such as biomedicine [29,30], industrial synthesis [31,32], and so on. When microfluidics is combined with engineering science and biomaterial science, diverse preparation techniques for multi-functional microspheres can be derived.

The development of microfluidic technology is inseparable from the progression of microfluidic instruments. In the flowing section, the microfluidic technology and its influence on the preparation of microsphere hydrogel will be introduced through different microfluidic devices.

#### 2.1.1 Microfluidic devices based on PDMS

Polydimethylsiloxane (PDMS) is a silicone polymer known for its excellent elasticity, biocompatibility, and chemical inertness. Its transparency allows it to be easily fabricated into pre-designed devices of various shapes and sizes through simple manufacturing processes. PDMS has been widely used in producing microfluidic chips, particularly for preparing hydrogel microspheres as drug carriers in its early applications [33].

**Fig. 1:**
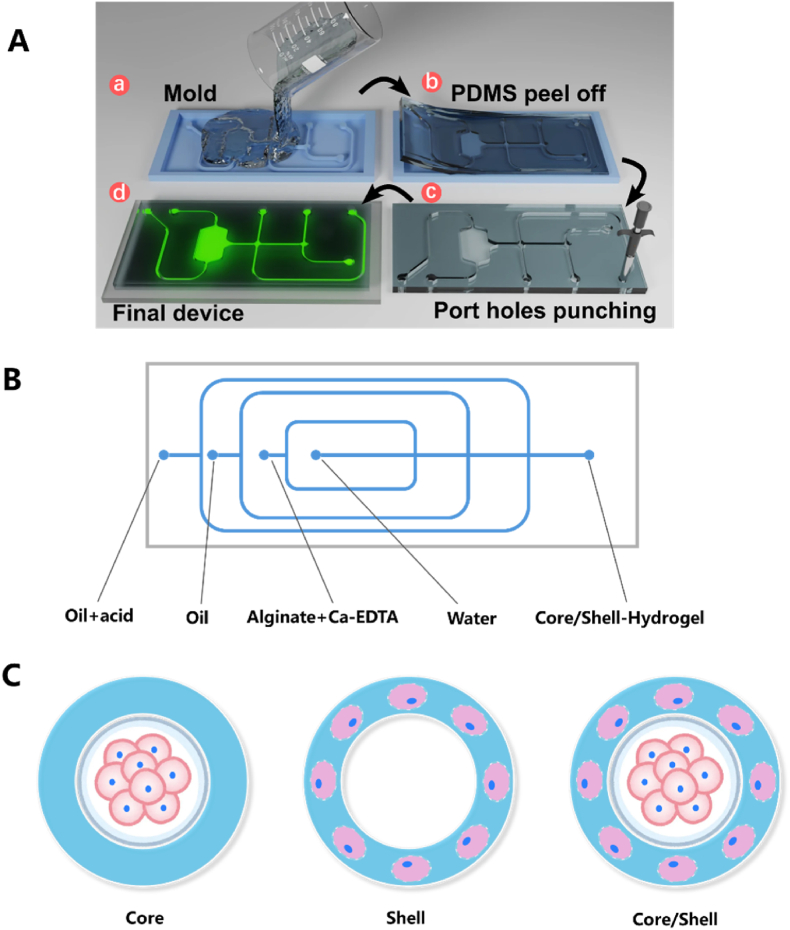
(A) The fabrication process of PDMS microfluidic chips: (a) Pouring PDMS onto the mold and allowing it to cross-link; (b) Peeling off the PDMS layer; (c) Puncturing fluid access holes; (d) Bonding the PDMS to a glass substrate and filling with fluorescein to enhance contrast. (B) A four-channel microfluidic chip device enables precise fluidic control. (C) Three-dimensional core-shell scaffolds facilitate the spatial organization and assembly of diverse cell types [39]. Copyright 2022, The Authors.

The preparation process typically follows these steps [[34], [35], [36], [37]]. First, master plates are created by UV etching with photoresist, followed by mixing PDMS and curing crosslinkers onto the master. After an overnight high-temperature curing treatment, the PDMS plate is detached from the mold, and the inlet and outlet are further opened. The PDMS plate is then bonded to a slide, and the channel is made hydrophobic (Fig. 1A). Furthermore, the chip produced by this method can accommodate multiple channels, in addition to the traditional two-phase separation dual channel, and can utilize multiple aqueous phases to meet the requirements of layered coating. The four-channel chip used by Chen et al. [38] is fabricated from PDMS-based microfluidic devices (Fig. 1B). In the chip, the inner phase consists of a medium with stem cells, which can interact with an alginate solution and a Ca-EDTA (ethylene diamine tetraacetic acid) solution to form microsphere droplets, in the downstream, oil containing acetic acid is introduced to promote calcium ion release, which then cross-links with two different carboxyl groups of alginates, forming a 3D network structure (Fig. 1C).

#### 2.1.2 Self-made capillary microfluidic device

Compared to PDMS soft lithography, homemade microfluidic chips are better suited for laboratory use due to their advantages in droplet control. One widely used type is the coaxial capillary microfluidic device, which is also simple to prepare [[40], [41], [42]]. In this method, a glass capillary tube is stretched to achieve a specific inner diameter. After surface treatment, it is inserted into another capillary tube, and the outer space is sealed and fixed using materials such as a dispensing dropper, epoxy resin, or other appropriate methods. This chip is particularly effective for producing high-throughput, monodisperse microspheres. However, due to its simple oil-in-water structure, it can only produce hydrogel microspheres made from a single material and cannot be used for the preparation of layered, multiphase complex hydrogels.

**Fig. 2:**
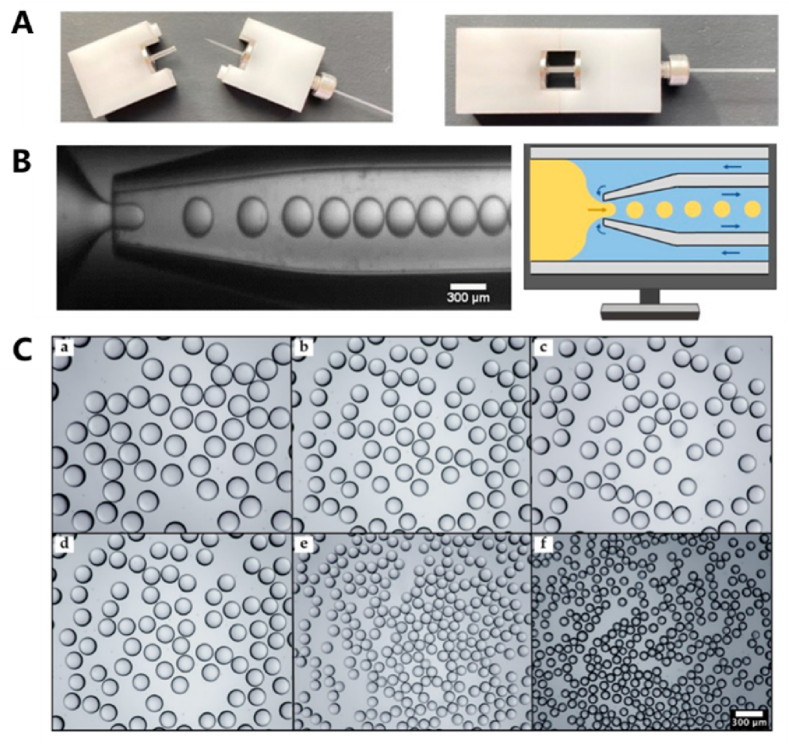
(A) A glass capillary device assembled from LEGO bricks. (B) The principle and schematic diagram of droplet formation process at the microscopic scale. (C) PEGDA particles generated by utilizing the inner capillary with diverse orifice diameters: (a) 400 μm; (b) 350 μm; (c) 300 μm; (d) 250 μm; (e) 200 μm; and (f) 150 μm. Reproduced with permission [43]. Copyright 2022, MDPI.

Chen et al. [43] and Yan et al. [44] developed ingenious method combining LEGO bricks and capillary tubes to create a specialized microfluidic device (Fig. 2A and B). To prepare the microfluidic chip, capillary tubes were stretched to the desired orifice size, polished, and treated with hydrophobic materials. The tubes were then installed in the middle of LEGO brackets using fasteners. By controlling the pore diameters, ranging from 150 to 400 μm, highly uniform and non-aggregating particles were produced, with an average size ranging from 107 to 239 μm. (Fig. 2C).

#### 2.1.3 Other microfluidic chips

**Fig. 3:**
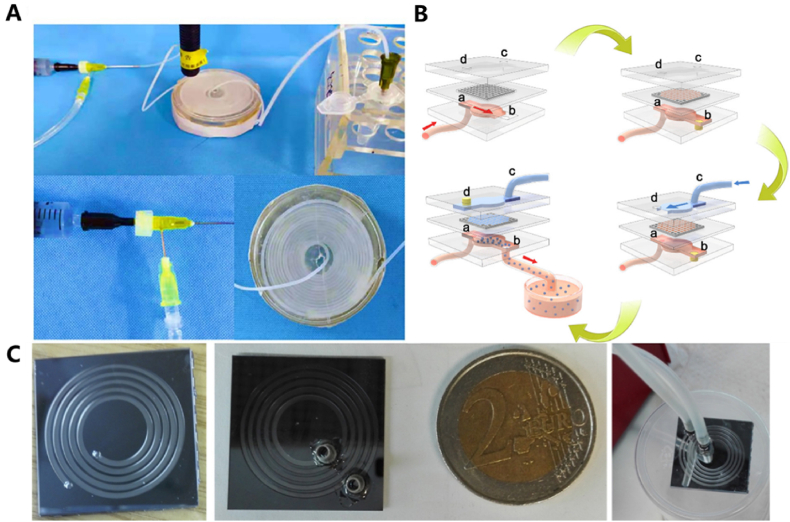
(A) A microfluidic manufacturing device for a syringe with coaxial placement. (B) The MCP membrane is like a sieve. The dispersed phase is the material that needs to pass through the sieve (the membrane). The pressure created by blocking the ports is similar to the force you would apply to push the material through the sieve. The continuous phase in the lower channel is like the container that catches the filtered material and combines with it to form the final product, which is the emulsion. Use a container to collect droplets from port b for further processing. (C) Fabricated silicon wafer microchannel reactor. Reproduced with permission [[47], [48], [49]]. Copyright 2020, Elsevier B.V. Copyright 2017, Elsevier B.V. Copyright 2023, American Chemical Society.

In addition to the two commonly used chips mentioned above, there are also specialized chips designed for specific applications, such as 3D printing [34,35], syringe connections [[45], [46], [47]], chemical etched silicon chips [48], and channel template [49]. For example, Wu et al. [47] designed a special GelMA solution generation device by placing two syringe needles of different thicknesses coaxially and connecting them with glue. The external part of the chip was connected to a plastic catheter, which was surrounded by a disc-shaped path with a diameter of 10 cm. UV light was applied to the disc to trigger the cross-linking and reverse curing of the solution. Hydrogel microspheres were collected using a collection device (Fig. 3A).

In another work, Wu et al. [49] employed a microchannel plate (MCP) as a high-throughput dispersal chip component to provide a dense capillary array over a large area. The subchannels were assembled in a molded PDMS template. A low-viscosity continuous phase was selected to ensure the resulting microspheres exhibited high uniformity and stability. During operation, the continuous phase flows from the lower side to fill the entire channel plate, followed by the injection of the dispersed phase from the upper channel. The dispersed solution enters the MCP, where it is distributed and subsequently collected from the lower outlet (Fig. 3B).

Ozlem et al. fabricated a coin-sized microfluidic chip using Pyrex glass and silicon wafers with a thickness of 500 μm [48]. The microchannels on the glass and silicon wafers were created using chemical etching and deep reactive ion etching respectively, and then connected (Fig. 3C). This method allows for the preparation of sodium alginate gels both with and without chitosan.

### 2.2 Emulsion method

**Fig. 4:**
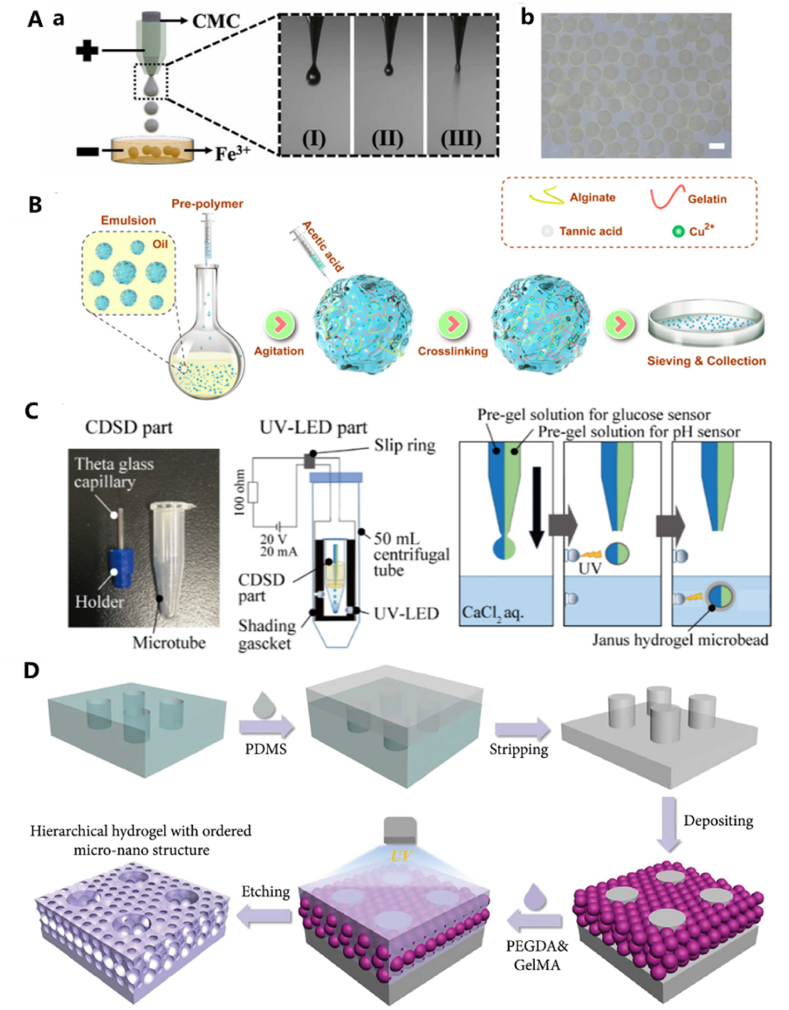
(A) Electrospray patterns and images showing particle formation under different voltages. (B) Diagram of preparation of microspheres by emulsion method. (C) Preparation of glucose and PH dual response Janus microspheres by UV-assisted centrifugation. (D) Schematic diagram of preparation of multistage hydrogels with ordered micro-nano structures. Reproduced with permission [51,52,54,57]. Copyright 2022, Elsevier B.V. Copyright 2024, American Chemical Society. Copyright 2021, MDPI. Copyright 2021, Science Partner Journals.

The emulsion method, first applied in the 1970s for microsphere preparation, is commonly used for batch production due to its relatively simple operation [50]. In this method, the aqueous solution is first suspended in an oil-based solvent, and then the solution is gelled in the aqueous phase using appropriate methods (such as chemical cross-linking, solution displacement, etc.) to finally form hydrogel particles. This method offers clear advantages in the batch preparation of hydrogel microspheres. However, the particle size of hydrogel microspheres prepared using this method can not be precisely controlled, the oil phase requires the use of organic solvents (such as mineral oil and cyclohexane) and surfactants (e.g., Span 80). Incomplete post-treatment may lead to cytotoxicity or inflammatory responses. Shen et al. synthesized SGTC antibacterial microspheres [51] by mixing sodium alginate, gelatin, and other materials using this method. Paraffin oil received the aqueous phase in a 2:3 ratio, and after stirring and cooling, acetic acid was added. The mixture was then subjected to centrifugation, washed with ethanol and purified water, and the samples were screened and collected. The microspheres produced in this way exhibited a typical particle size distribution between 100 and 400 μm, along with good porosity and water absorption properties (Fig. 4B).

The “Nitrogen scavenging method” used by Xiong et al. [52] can also be considered a type of emulsion method. The preparation of microspheres involved expelling droplets of a 2 % ALG solution from a syringe, a nitrogen stream, with a flow rate of 15 L/min, was then directed from the top to the bottom above the needle to break large droplets into smaller ones. These smaller droplets subsequently fell into a Petri dish filled with NiCl_2_ solution, forming hydrogel microspheres. After being collected by centrifugation, the Ni-ALGMS hydrogel microspheres can be evenly dispersed in either phosphate-buffered saline (PBS) or water for long-term storage. Ni-ALGMS appear as spherical or pebble-like structures with a diameter ranging from 50 to 200 μm.

### 2.3 Electrospray

Electrospray, also referred to as microfluidic electrospray [53,54], is a method in which a liquid sample is stretched using the electric field generated by the device. Since its inception, electrospray technology has evolved from fundamental explorations of ionization principles to widespread applications. By leveraging high-voltage electric fields to atomize solutions into microdroplets and subsequently solidify them into microparticles, electrospray has achieved significant advancements in the fields of drug delivery, biomedicine. When the voltage is increased to a certain level, the solution forms tiny droplets of uniform size, and hydrogel microspheres are ultimately formed by the gelation of the collected droplets. In the conventional electrospraying method, the process does not require a microfluidic chip; the spray is generated directly through the application of a high electric field at the needle tip. However, integrating a microfluidic chip into the electrospraying system offers precise control over liquid delivery and flow rates. The electrospray technique demonstrates distinct advantages in hydrogel microsphere fabrication. Precise control over microsphere dimensions (typically ranging from micron to nanoscale) can be achieved through systematic parameter optimization, including electric field strength, solution flow rate, and nozzle diameter. This method operates under mild processing conditions (ambient or low temperatures), effectively preserving the bioactivity of thermosensitive components such as proteins and enzymes without requiring harsh chemical treatments. Notably, the implementation of physical crosslinking strategies (e.g., temperature-responsive gelation or ionic crosslinking) or photo-curing mechanisms eliminates the need for chemical crosslinkers, thereby significantly reducing potential biotoxicity risks associated with residual reagents. Despite these merits, several technical challenges persist. The process inherently relies on specialized equipment such as high-voltage power supplies, precision nozzles, and environmental control systems, resulting in substantial initial capital investment and maintenance costs. While single-nozzle configurations exhibit limited production capacity, scaling up through multi-nozzle arrays may introduce inter-electrode interference, potentially compromising size uniformity and hindering industrial-scale manufacturing efficiency. Furthermore, microsphere collection faces challenges including electrostatic adhesion and premature aggregation due to incomplete crosslinking, necessitating advanced collection apparatus optimization (e.g., liquid bath systems) or post-processing techniques to ensure product integrity [55,56]. As an illustration, Zhu et al. [55] prepared a capillary device by drawing and grinding capillary tubes to 250 μm. By controlling the voltage and flow rate, fish skin gelatin microspheres were produced. The results also demonstrated that the particle size has a direct proportional relationship with the voltage and an inverse proportional relationship with the liquid flow rate. The size of the hydrogel microspheres remained uniform and stable within the range of 350–550 μm using this method (Fig. 4A).

### 2.4 Mechanical crushing

Mechanical stirring methods, primarily relying on conventional stirring apparatus and originating from basic principles of macroscopic shear force application, have evolved through continuous optimization of mixing parameters and integration with other techniques to enhance their applicability and uniformity in particle size control, despite inherent limitations in precision. which significantly reduces capital expenditure and operational complexity. Ion crosslinking, photo-crosslinking, and chemical crosslinking are among the various gelation approaches employed. However, achieving submicron or monodisperse microspheres (<50 μm) is challenging. Compared with microfluidic or membrane-based methods, mechanical forces are less effective in generating uniform small droplets. Moreover, additional steps such as solvent extraction, crosslinking bath immersion, and repeated washing are typically required to remove residual oil phase and surfactants, thereby increasing process duration and the risk of contamination. Anh et al. prepared methacrylate gelatin particles using this method [58]. The preparation process was as follows: a mixture of corn oil and solution was homogenized by centrifugation, followed by stirring to initiate the thermal cross-linking reaction at high temperature. The mixture was then cooled and washed by centrifugation, allowing the excess oil to be removed and the hydrogel particles to be obtained. The results showed that the microspheres prepared under different conditions using this method generally have a smooth, circular shape, though most of them fuse together, resulting in poor dispersion, which makes it difficult to evaluate their individual morphology.

### 2.5 Other methods

In addition to the widely used methods for preparing hydrogel microspheres, several other techniques have been explored, such as the mesoporous silica template method [13], UV-assisted centrifugation [57], suspension polymerization and spray drying. Ando et al. [57] prepared hydrogel microspheres by fixing cut and elongated capillaries to a scaffold that could be rotated and spraying the prepolymer solution onto them using a UV-LED device below to complete the gelation process. (Fig. 4C). Zhu et al. [59] fabricated a multistage hydrogel with ordered micro-nanostructures and achieved a smooth, complete PDMS microarray by replicating a PMMA template. Silica-containing nanoparticles in an ethanol solution were introduced onto the PDMS microarray. Following ethanol evaporation, SiO_2_ nanoparticles self-assembled on the PDMS microarray. A pre-gel mixture of polyethylene glycol diacrylate (PEGDA) and methacrylate gelatin (GelMA) was then filled into the gaps between the nanoparticles and the microarray. After ultraviolet (UV) light-induced photopolymerization, the microspheres were collected (Fig. 4D).

Spray drying involves atomizing a solution containing polymer precursors into fine droplets, which are then rapidly dehydrated and solidified by a high-temperature airflow, resulting in the formation of microspheres. This method is particularly effective for natural polymers with good thermal stability, such as starch and gelatin, or for systems that undergo rapid crosslinking [60]. On the other hand, suspension polymerization is a technique where water-soluble monomers are dispersed as droplets within a non-polar oil phase and polymerized under the influence of an initiator. The droplets remain stable due to the stabilizing effects of surfactants and mechanical agitation, leading to the formation of crosslinked hydrogel microspheres. This method is especially suitable for hydrophobic or amphiphilic monomers. Microsphere size can be controlled by adjusting parameters such as stirring speed and the oil-to-water phase ratio, and the process is similar to emulsion polymerization [61].

Each hydrogel microsphere preparation method has its own set of advantages and disadvantages. Microfluidic droplet technology offers precise control over microsphere size and shape, allowing for high reproducibility and uniformity, but it may be limited by scalability and the complexity of device design. Emulsion methods are versatile and widely used, providing good control over particle size, but they can require surfactants, which might affect biocompatibility. Electrospray enables the production of small, uniform microspheres with high throughput, but it may struggle with viscosity limitations and requires high electrical fields. Mechanical crushing is simple and cost-effective, though it can produce irregular sizes and lacks fine control over particle characteristics. The mesoporous silica template method offers excellent control over microsphere porosity and size, but the process is time-consuming and requires additional steps. UV-assisted centrifugation allows for fast and efficient microsphere formation, particularly for crosslinking sensitive materials, but it may require specialized equipment and may not be as scalable. Suspension polymerization and spray drying are both efficient methods for producing large quantities of microspheres, with suspension polymerization offering good control over the size of microspheres and spray drying being useful for creating microspheres from thermally stable polymers, though both methods can suffer from challenges in achieving uniformity and controlling morphology. Each method has its trade-offs, and the choice depends on the specific requirements of the application, including factors like size control, material compatibility, and scalability.

## 3 Biofunctionalization strategies of hydrogel microspheres

Hydrogel microspheres are characterized by excellent stability, injectability, and the ability to uniformly encapsulate molecules and cells, allowing for sustained and controlled release. These properties make them highly promising for a wide range of biomedical applications. To further maximize their potential, hydrogel microspheres are often functionalized to impart distinctive properties tailored to specific biomedical needs. Functionalization strategies primarily modify the physicochemical characteristics of hydrogels, enabling them to respond to specific physiological or pathological conditions, thus facilitating targeted therapy or intelligent drug delivery. This can be achieved through approaches such as surface modification with targeting molecules, antibody or ligand conjugation, and the integration of pH or enzyme-responsive mechanisms.

At the same time, the crosslinking method plays a crucial role in determining the functional properties of hydrogel microspheres, directly influencing their biological activity and application performance. Different crosslinking strategies impart distinct mechanical properties, degradation behaviors, and bioactivities to the hydrogel network. For instance, chemical crosslinking allows fine-tuning of mechanical strength and biodegradation rates by adjusting crosslinking density, making it particularly useful for applications requiring long-term structural integrity. However, excessive chemical crosslinking may lead to reduced biocompatibility due to residual crosslinking agents or increased stiffness, which can hinder cell migration and proliferation. In contrast, physical crosslinking, such as ionic or hydrogen bonding interactions, offers improved biocompatibility and dynamic reversibility, enabling hydrogels to respond to environmental stimuli, though often at the cost of reduced mechanical stability. Photopolymerization provides precise spatial and temporal control over crosslinking, allowing on-demand gelation, which is beneficial for in situ applications such as biofabrication and minimally invasive therapies [62].

To enhance the functional properties of hydrogel microspheres, optimizing crosslinking strategies is essential. Selecting appropriate crosslinking agents, fine-tuning crosslinking density, and incorporating stimuli-responsive elements can significantly improve biological activity. For example, hydrogels modified with integrin-binding peptides or bioactive crosslinkers can enhance cell adhesion, proliferation, and differentiation, thereby improving their performance in tissue engineering and regenerative medicine. Moreover, dual-crosslinking approaches, which combine covalent and non-covalent interactions, can balance mechanical stability with dynamic adaptability, enabling hydrogels to better mimic the extracellular matrix and facilitate controlled drug or gene delivery [63].

By systematically optimizing crosslinking methods, hydrogel microspheres can achieve superior functionalization, paving the way for more effective and versatile applications in precision medicine, tissue engineering, and regenerative therapies.

### 3.1 Cell loading

Hydrogels with a three-dimensional network exhibit extracellular matrix-like properties, making them ideal carriers for bioactive substances. Hydrogel microspheres are commonly used to deliver bioactive agents for specific purposes. Cell delivery is one of the most prevalent methods for functionalizing hydrogel microspheres. Various cell types can be encapsulated within the microspheres to serve different functions, either by co-gelation with a solution during microsphere preparation using a microfluidic device [[64], [65], [66], [67]], or by curing the microspheres first and then attaching the cells. Both approaches are important for effective cell delivery [68].

#### 3.1.1 Cell encapsulation

**Fig. 5:**
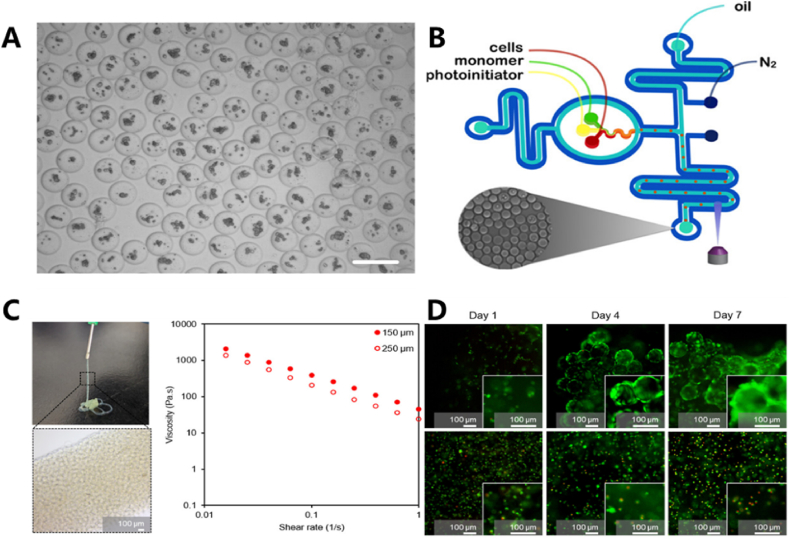
(A) Encapsulation of mESCs with PEG hydrogel microspheres. (B) Schematic diagram of a two-layer nitrogen jacket microfluidic device. (C) The viscosity of the hydrogel derived from tilapia decreases with the increase of shear rate. (D) Live/dead fluorescence was performed on 3D cell cultures of L929 cells in dECM particle hydrogel assemblies and dECM bulk gel for 7 days. Reproduced with permission [[69], [70], [71]]. Copyright 2016, Elsevier B.V. Copyright 2016, American Chemical Society. Copyright 2024, American Chemical Society.

Cell encapsulation is a technique used to functionalize various materials by embedding cells within them. Hydrogel microspheres with stable structures and low biotoxicity are ideal candidates for cell encapsulation. Various types of cells can be encapsulated in the microspheres and cultured through processes of cell differentiation and proliferation. Ana et al. [66] achieved cell growth by encapsulating human bone marrow-derived mesenchymal stem cells (hMSCs) in PEG hydrogel microspheres. The hMSCs were collected from bone marrow and cultured for 2–5 generations, followed by digestion, centrifugation, and precipitation, the cells were then suspended in prepolymer at a density of 1.2 × 10^6^ cells/mL to prepare PEG-based hydrogel microspheres. The results demonstrated the cell and metabolic activity of PEG-4aNB microgels at different concentrations. As the incubation time increased, higher hydrogel concentrations correlated with higher metabolic activity. Similarly, Siltanen et al. suspended multipotent embryonic stem cells (mESCs) in a solution containing 10 % 8-arm PEG-thiol, 4 % w/v PEGDA, and 7 % w/v heparin-MA for cell encapsulation. The hydrogel particles were generated with diameter exceeding 200 μm using a microfluidic focusing device [69]. On average, each gel droplet encapsulated 12 ± 3 cells, and the viability of the encapsulated mESCs exceeded 95 % (Fig. 5A).

The preparation of microspheres by mixing the solution helps protect cells from external mechanical stress. Several factors, including crosslinking agents and the oxygen content in the solution, can influence cell survival rates. Xia et al. introduced a nitrogen micro-jacketed microfluidic platform for preparing hydrogel microspheres with encapsulated cells. By using this device, oxygen solubility in both fluorinated oil and prepolymer solutions was effectively reduced by purging nitrogen through the microfluidic channels, thereby preventing the formation of reactive oxygen species (ROS) during photo-triggered polymerization of PEGDA. Additionally, the study simulated the generation of peroxy free radicals under varying initiator concentrations and UV intensities, all of which significantly affected cell viability after encapsulation. The results demonstrated that the nitrogen micro-jacketed microfluidic platform could fully restore cell viability. (Fig. 5B) [70].

#### 3.1.2 Cell loading on surface

Another method for modifying hydrogel microspheres with cells is to co-mix the microspheres with cells during loading. This approach eliminates concerns about potential cell damage during the gelling process and helps better maintain the survival rate of treated microspheres when co-cultured with cells. Several biocompatible materials, such as gelatin and acellular materials, are commonly used to prepare hydrogel microspheres for cell loading. Pilseon et al. prepared hydrogel microspheres for cell loading using decellulated tilapia skin [71]. In this study, the decellularized extracellular matrix (dECM) hydrogel microbeads were assembled into 3D scaffolds using hyaluronic acid-catechol (HA-catechol) via microfluidic flow focusing technology. Specifically, 4.2 mL of dECM microgel was mixed with 1.0 × 10^7^ L929 cell dispersion in 0.8 mL of 2 wt% HA-catechol to prepare the dECM particle hydrogel components. The dECM particle hydrogel was applied to the surface of pig skin and retained adhesion even when subjected to forces from all directions. In the case of dECM particle hydrogel assembly, rapid formation of cell networks occurred by day 4, and continuous cell proliferation was observed across the entire surface of the hydrogel scaffold, with high cell survival remaining by day 7 (Fig. 5C and D).

### 3.2 Functional metal oxide loading

Nanoparticles are small particles within the nanometer size range, exhibiting unique physical, chemical, and surface properties. They are commonly used to modify both organic and inorganic materials, playing a significant role in enhancing the functional properties of hydrogel microspheres. In the context of hydrogel microsphere preparation and functionalization via microfluidic methods, nanoparticles are extensively utilized. Depending on their composition, nanoparticles can be broadly classified into metal-based nanoparticles [[72], [73], [74]], organic nanoparticles [75,76], nano-enzyme particles [77], and composite nanoparticles [[78], [79], [80]]. Due to their exceptional properties, nanoparticles have significantly enhanced the characteristics of hydrogel microspheres. Among the various types, metals, metal oxides, and their composites are commonly used to prepare functional nanoparticles, which may also be used to endow hydrogel microspheres with unique functions. Yang et al. [72] fabricated microspheres containing Cu ions, which exhibited good antibacterial properties and angiogenic effects due to the continuous release of copper ions. In this study, the microspheres were prepared using the electrospray method, Carboxymethyl cellulose (CMC) and gelatin methylacrylamide (GelMA) droplets were mixed, collected in a Cu(NO_3_)_2_ solution, and cross-linked under UV irradiation for 5 min. Lin et al. developed a monodisperse core-shell microsphere transport system composed of poly(lactic-co-glycolic acid) (PLGA) biopolymer and magnesium oxide (MgO) nanoparticle-loaded alginate [81]. The core of the hydrogel microsphere facilitated the transport and release of magnesium ions. In another study, Zhang et al. [79] prepared Fe_3_O_4_ nanoparticles via a hydrolysis reaction, then coated them with polydopamine (PDA), graphene oxide (GO), and a SiO_2_ coating. The multiple responsive agarose microcarrier was generated via water-in-oil method using a PDMS microfluidic device. In addition to the magnetic responsiveness of Fe_3_O_4_, the GO component also exhibited excellent photothermal response under near-infrared (NIR) radiation. DNA detection using these microcarriers demonstrated good specificity, linearity, and accuracy.

### 3.3 Bioactive molecule loading

**Fig. 6:**
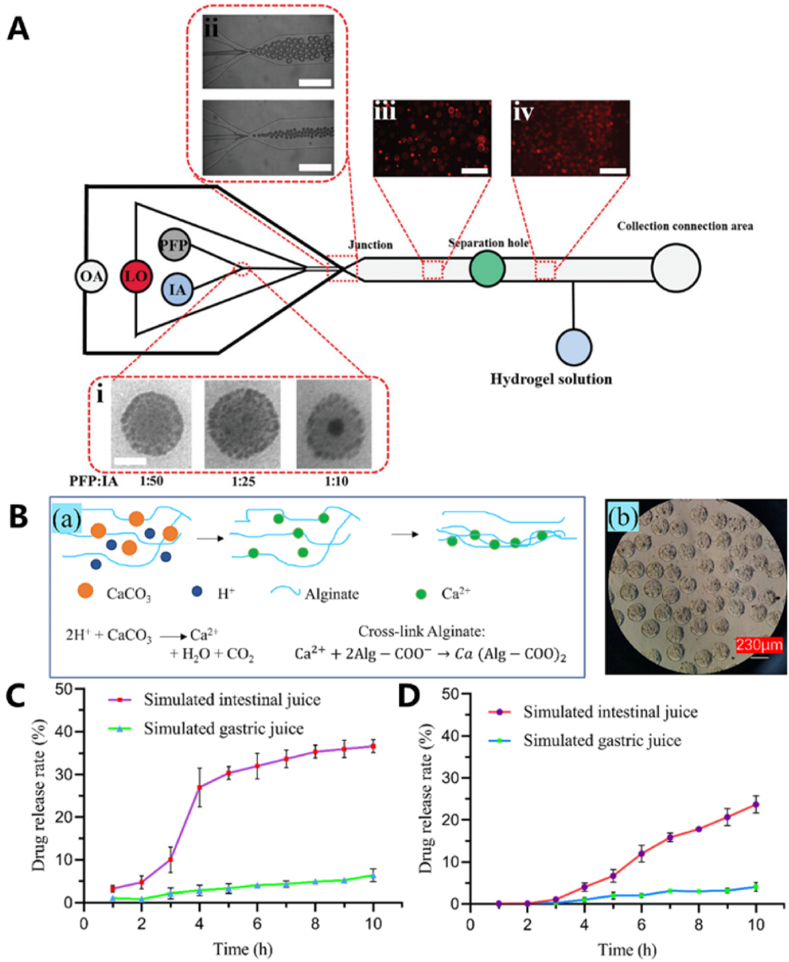
(A) Formation of U-CMLV hydrogel microspheres (B) Alginate saline gelatin crosslinking process diagram and prepared hydrogel microspheres (C) Single-layer hydrogel microcapsules in simulated gastric fluid and simulated intestinal fluid for drug release (D) Core-shell hydrogel microcapsules in simulated gastric fluid and simulated intestinal fluid for drug release. Reproduced with permission [84,85]. Copyright 2023, John Wiley and Sons. Copyright 2024, Elsevier B.V.

Bioactive molecules can be categorized based on their source, function, and chemical structure, including natural products, synthetic compounds, immunomodulatory molecules, antioxidant molecules, and antimicrobial molecules [82]. These molecules play a critical role in tissue engineering and are essential for the effective diagnosis, treatment, and repair of tissues. Their efficient transport and controlled release are key factors in optimizing therapeutic outcomes. Hydrogel microspheres serve as an ideal delivery system, enhancing the transport, stability, and bioactivity of bioactive molecules, which makes them widely used in drug carrier design, controlled release, and targeted delivery [83]. He et al. obtained ultrasound-controlled monodisperse lipid vesicles (U-CMLVs) using microfluidic technology [84]. The internal aqueous phase consisted of a solution containing an ultrasonic reactive substance (perfluoropentane, PFP), drugs, fluorescent substances, and other components, this solution was sheared through a liposomal organic phase to form a W/O droplet. The external water phase, flowing through the shear process, formed dispersed W/O/W droplets. The organic layer was automatically separated from the W/O/W droplets in subsequent channels, then fused with the external GelMA hydrogel solution after passing through the chip. Finally, the mixture solidified into gel via ultraviolet irradiation (Fig. 6A). When the drug was released, the U-CMLVs could be ruptured by adjusting the frequency of ultrasound waves from high to low to achieve controlled drug delivery.

Traditional drug therapy merely delivers drugs into the body but lacks the ability to target and selectively direct them to diseased tissues, resulting in relatively low drug delivery efficiency. Localized drug delivery using microspheres, on the other hand, can increase the concentration of the drug at the affected tissues, thereby improving delivery efficiency and achieving better therapeutic outcomes. Qiao et al. [85] developed a single-layer, pH-responsive hydrogel microcapsule loaded with indomethacin. By adjusting the pH with normal saline, they simulated the gastric and intestinal fluid environments. The drug release from this liquid carrier gel was significantly lower in simulated gastric fluid with a low pH compared to that in simulated intestinal fluid, successfully demonstrating the effect of small intestinal-targeted drug delivery (Fig. 6B–D).

## 4 Biomedical applications of hydrogel microspheres

### 4.1 Biological sensing and detection

**Fig. 7:**
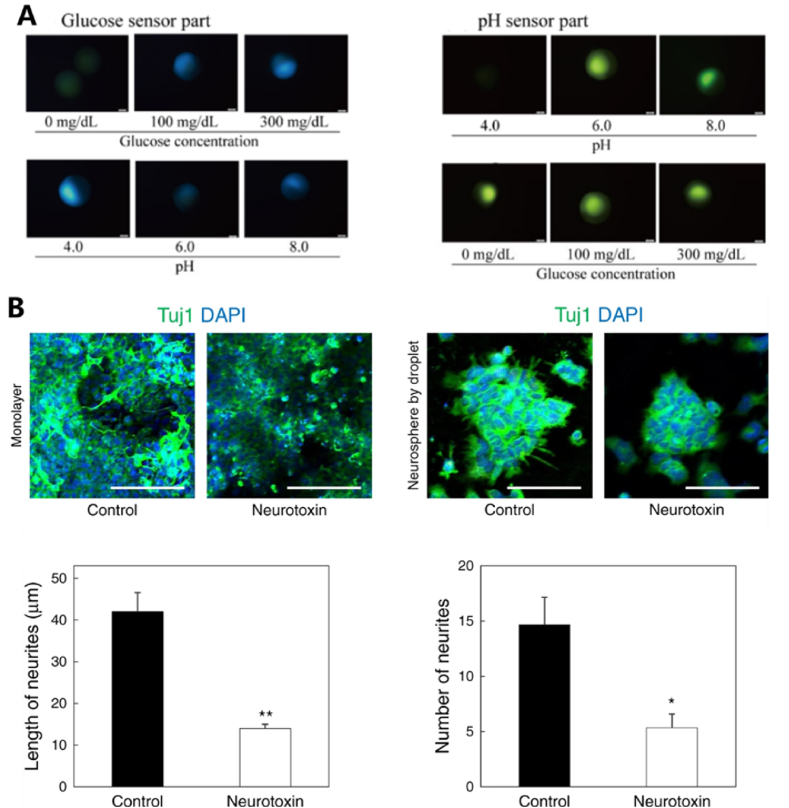
(A) Fluorescence images of glucose and pH probe components at various glucose concentrations and pH conditions. (B) Drug screening analysis for neurospheres produced by droplet-based microfluidic devices: Images obtained through confocal laser scanning microscopy of monolayer neurons and quantitative analysis regarding the length and number of neurites originating from the neurospheres. Reproduced with permission [57,87]. Copyright 2021, MDPI. Copyright 2020, Springer Nature Limited.

Hydrogel microspheres with molecular targeting capabilities can specifically recognize organs and tissues, demonstrating great potential in fields such as sensing, detection [86], drug screening [[87], [88], [89]] and more. Their high sensitivity to various chemical and biological signals further enhances their applicability. Ando et al. prepared multifunctional Janus hydrogel microbeads for pH sensing [57]. The hydrogel microbeads consist of two components: one acts as a glucose sensor, while the other serves as a pH sensor. These dual components impart fluorescence-responsive characteristics to the microspheres. The results showed that as the glucose concentration increased, the fluorescence intensity of the glucose sensor also increased. Meanwhile, when the pH value rose, the fluorescence intensity of the pH sensor also increased (Fig. 7A). The authors suggested that introducing a three-chamber microbead design, with a fluorescence reference chamber, could improve the accuracy of fluorescence intensity measurements, thereby enabling more accurate and stable blood glucose monitoring in the future.

MicroRNA is a class of non-coding RNA sequences composed of 20–25 nucleotides, which exert a significant regulatory function within the cell and get involved in the process of gene expression, cell multiplication and differentiation. Mazzarotta et al. designed functional hydrogel particles for miRNA detection [90], focusing on MiR-143–3p, a biomarker with significant clinical value. MiR-143–3p had been shown to play a crucial role in the diagnosis and prognosis of various diseases. Its detection was facilitated by optimizing the structural parameters of 3D-PEG hydrogel particles, using double-strand displacement within the particles to enable target recognition. The method demonstrates a low detection limit. Additionally, the experiments showed that the hydrogel microparticles possess molecular filtration and detection capabilities, enabling them to select target molecules of appropriate size and detect them directly in complex liquids. This approach holds great promise for non-invasive screening, as well as the early detection and monitoring of multiple diseases.

Lee et al. developed a highly efficient in vitro evaluation method using innovative microfluidic techniques to generate tumor spheres with uniform size and structure, integrating drug screening and photothermal therapy [87]. The results showed that thapsigargin treatment significantly reduced the Tuj1 intensity in the monolayer of neurons. Additionally, the authors observed neuronal damage in the neurospheres following thapsigargin treatment, with the average neurite length decreasing from 42 μm to 14 μm and a significant reduction in the number of neurites. Therefore, this droplet-based microfluidic device is suitable for drug screening in various neurological diseases (Fig. 7B).

### 4.2 3D cell culture

**Fig. 8:**
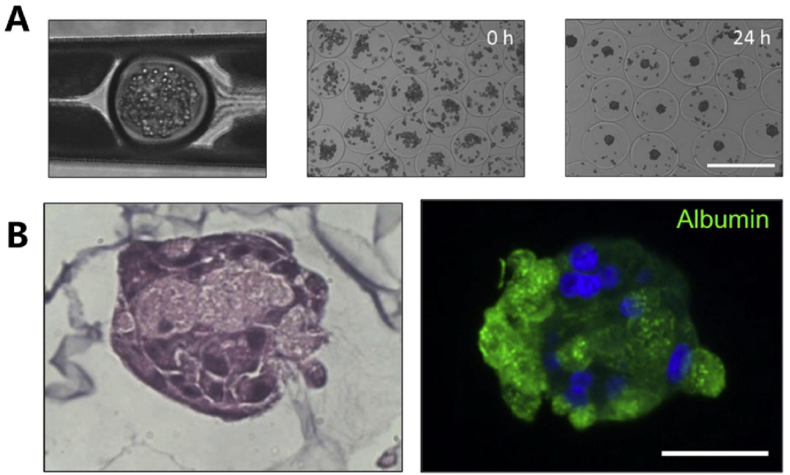
(A) DTT induced crosslinked cell capsules were prepared in a microfluidic device, and hepatocytes were immediately encapsulated in aqueous medium after collection. After 24 h, the clustered cells come together to form compact globules. (B) H&E histology and immunofluorescence staining confirmed the presence of intracellular albumin in the encapsulated hepatic spheres. Reproduced with permission [91]. Copyright 2017, Elsevier B.V.

Hydrogel microspheres with stable structure and low toxicity are excellent candidates for cell culture. Micro-sized hydrogels, serving as supports for three-dimensional cell culture, offer a platform that mimics the tissue microenvironment, enabling a more realistic simulation of cell growth and function in vivo. Siltanen et al. presented a simplified approach to encapsulate cells within PEG microcapsules [91]. This approach combined microsphere fabrication, hepatocyte encapsulation, and cell aggregate formation. Rheological and transport analyses revealed that large serum proteins could be exchanged through the capsule shell under all tested conditions, ensuring the adequate exchange of soluble factors or gases, nutrients, and wastes secreted or accumulated by the cells (Fig. 8A and B).

Hwang et al. [92] fabricated a three-dimensional cell-laden alginate hydrogel with controllable porosity by leveraging the thermo-responsive gelation properties of gelatin. This hydrogel demonstrated an approximately two-order-of-magnitude increase in permeability and exhibited superior performance in terms of cell proliferation, cell aggregate formation, and albumin secretion. These features suggest promising applications in the field of tissue engineering. Cheng et al. reported a biocompatible GelMA hydrogel microsphere. They found that hydrogel microspheres with 10 % (w/v) GelMA showed high porosity and was convenient for the exchange of nutrients required by cells [93]. To address the issue of low cell aggregation and survival in current cell transplantation strategies, a double-network crosslinked hydrogel microsphere, named GCMS, was synthesized by incorporating 1 % (w/v) chitosan as a carrier for ARPE-19 cell transplantation.

### 4.3 Tissue repair

#### 4.3.1 Wound healing

**Fig. 9:**
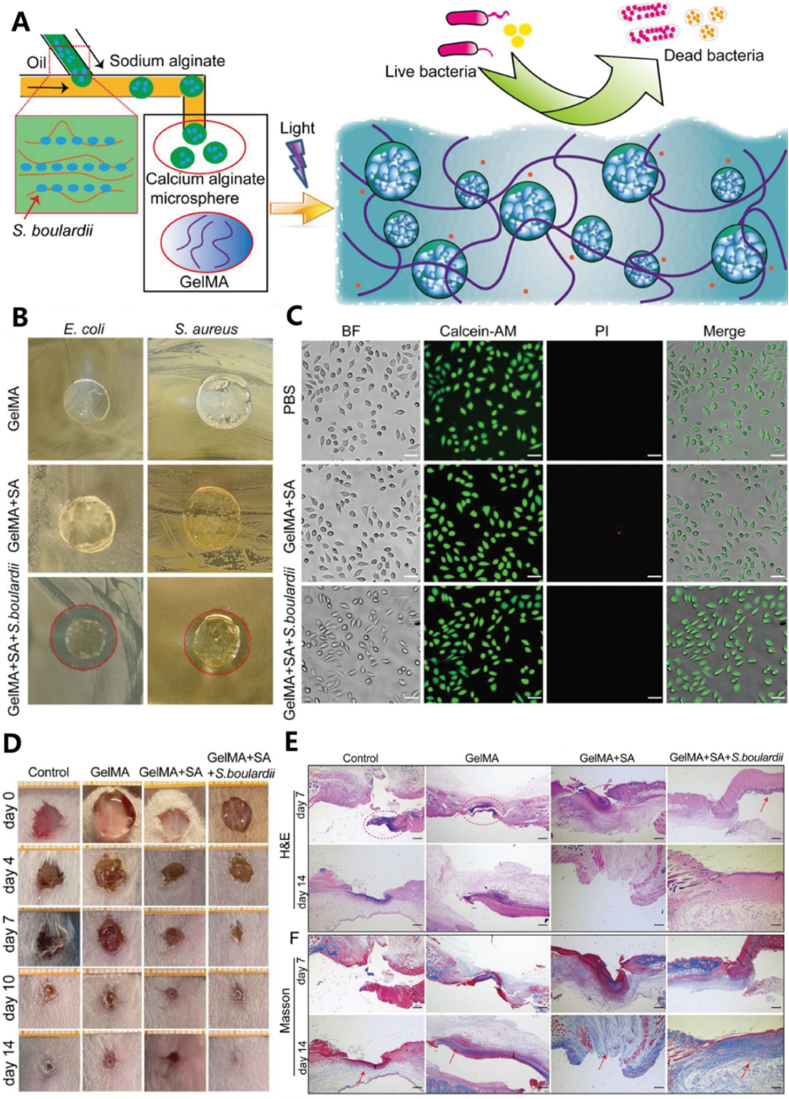
(A) microfluidic method encapsulated Streptococcus bullae in alginate microspheres and prepared an active probiotic dressing. (B) Antimicrobial test of active probiotic dressings against *Escherichia coli* (*E. coli*) and *Staphylococcus aureus* (*S. aureus*). (C) Biocompatibility test of active probiotic dressings. (D) Wound healing of *Staphylococcus aureus* (*S. aureus*) infection in mice. (E) Hematoxylin and eosin (H&E) Masson tri-color dyeing. Reproduced with permission [98]. Copyright 2023, John Wiley and Sons.

Tissue damage is a prevalent and challenging issue in both daily life and clinical medicine, and tissue healing is a complex process. During wound healing, infection is common, and infection control significantly influences the wound repair process [94]. Hydrogel microspheres can be used to cover wounds, providing anti-inflammatory effects [95] and promoting wound healing [96,97]. *Saccharomyces boulardii* (*S.boulardii*), a clinically used probiotic, secretes antimicrobial substances and serves as a valuable and safe therapy for preventing bacterial infections. Wei et al. prepared a two-layered structure consisting of sodium alginate microspheres on the inside and photopolymerized GelMA hydrogel on the outside. The microspheres, containing probiotics, exhibited antibacterial properties, while the GelMA hydrogel layer not only prevented the probiotics from escaping but also contributed to the antibacterial effect [98]. Additionally, microcapsules containing hepatocytes were cultured on a fibroblast culture layer, and hepatocyte globules were separated from the cells with the aid of a hydrogel shell, which was 10 μm thick to further enhance liver function. Studies on *Staphylococcus aureus*-infected wounds in mice showed that hydrogels without *S.boulardii* exhibited lower wound healing rates than those with live probiotic dressings. After culturing bacteria isolated from infected wounds the following day, large amounts of bacteria were found in all groups except the experimental group, indicating significant bacterial growth (Fig. 9).

#### 4.3.2 Bone repair

Trauma, infection, and tumors contribute to a significant number of people suffering from bone deficiency-related diseases each year, including cartilage injury, osteoarthritis, and osteoporosis. Bone repair primarily involves the generation of new bone, which requires the differentiation of osteoblasts and the creation of an appropriate microenvironment. However, current treatment methods often fail to meet the diverse needs of bone tissue repair. Hydrogel microspheres, especially functionalized hydrogel microspheres, have shown promise in bone repair applications. Drug-loaded hyaluronic acid hydrogel microspheres have been found to promote the proliferation and differentiation of subchondral bone marrow mesenchymal stem cells (BMSCs) and allow for the visualization of in-situ cartilage regeneration [99]. Additionally, bisphosphonate (BP) grafted onto the surface of the microspheres, with the ability to capture Mg^2+^, can activate both osteoblasts and osteoclasts. These injectable hydrogel microspheres represent an effective approach for treating bone defects, particularly in osteoporosis models [100].

**Fig. 10:**
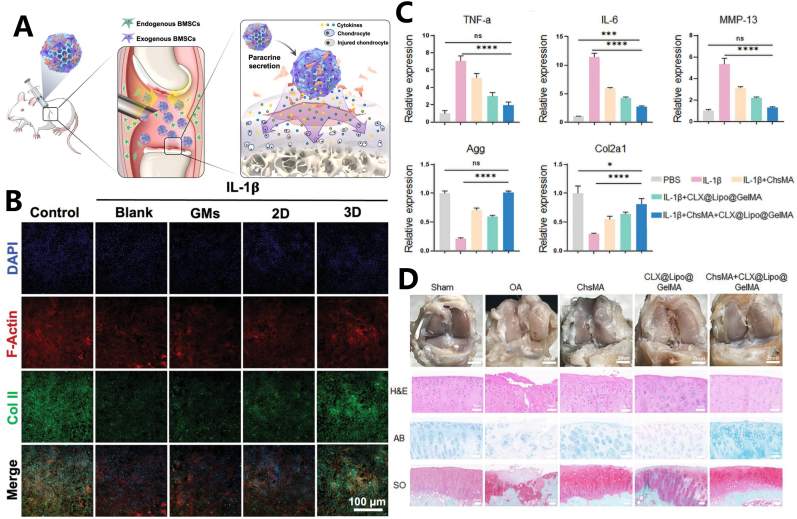
(A) GMs were injected into the joint cavity of DMM rats. The paracrine activity was enhanced by integrating the endogenous and exogenous regeneration mechanisms. (B) Immunofluorescence images of chondrocytes 48 h following IL-1β stimulation. (C) The mRNA expression levels of TNF-α, IL-6, MMP-13, Agg and Col2a1 of IL-1β-treated cartilage were analyzed by qRT-PCR when cultured with five types of cell hydrogel microspheres for 24 h. (D) ChsMA + CLX@Lipo@GelMA double-layer microspheres mitigated cartilage degeneration in OA rats 8 weeks post-surgery. Reproduced with permission [101,102]. Copyright 2024, John Wiley and Sons. Copyright 2024, Elsevier B.V.

Li et al. constructed porous GelMA hydrogel microspheres incorporating platelet-derived growth factor-BB (PDGF-BB) and exogenous mesenchymal stem cells (MSCs) [101]. The porous structure of the GelMA hydrogel microspheres extended the preservation time of cells at the defect site and facilitated interactions between the cells. The results demonstrated that continuous release of PDGF-BB in vivo attracted endogenous stem cells and enhanced their paracrine function (Fig. 10A), while also exhibiting optimal anti-inflammatory properties when the GelMA microspheres loaded with BMSCs were introduced into the upper chamber (Fig. 10B). In a separate study, Miao et al. developed a double-layer microsphere for treating osteoarthritis (OA) [102].

The microspheres, which respond to the microenvironment, were prepared using microfluidic technology. The outer layer of the double-layer microsphere was made of chondroitin sulfate methacryloyl (ChsMA) microspheres, which encapsulated celecoxib (CLX) in liposomes and were then soaked in a GelMA solution. In response to matrix metalloproteinase (MMP)-mediated degradation, GelMA degrades, releasing the CLX-loaded liposomes. The expression levels of TNF-α and IL-6 were significantly reduced, suggesting that CLX liposomes play an anti-inflammatory role in bone repair. As the internal microspheres degrade, chondroitin sulfate is gradually released, the significant increase in mRNA expression of Aggrecan (Agg) and Collagen type II (Col2a1), both critical components of the extracellular matrix involved in chondrocyte metabolism and synthesis, indicates that the treatment promotes chondrocyte anabolism and cartilage repair (Fig. 10C). Morphological evaluation of the knee joints in rats revealed that, compared to the Sham group, the articular cartilage surface in the hydrogel microsphere treatment group showed effective healing and a smooth, normal appearance (Fig. 10D) [103].

#### 4.3.3 Cancer therapy

Cancer poses a significant global health challenge due to its high incidence and mortality rates. The treatment of cancer remains a critical area of medical research and application. Efficient drug delivery is central to effective cancer treatment, as it directly impacts therapeutic outcomes. Consequently, innovative drug delivery systems have become increasingly important in cancer therapy [104], as they can minimize damage to healthy tissues and reduce side effects. Hydrogel microspheres, with their targeted and long-acting controlled release capabilities, have garnered significant attention as promising vehicles for cancer drug delivery.

**Fig. 11:**
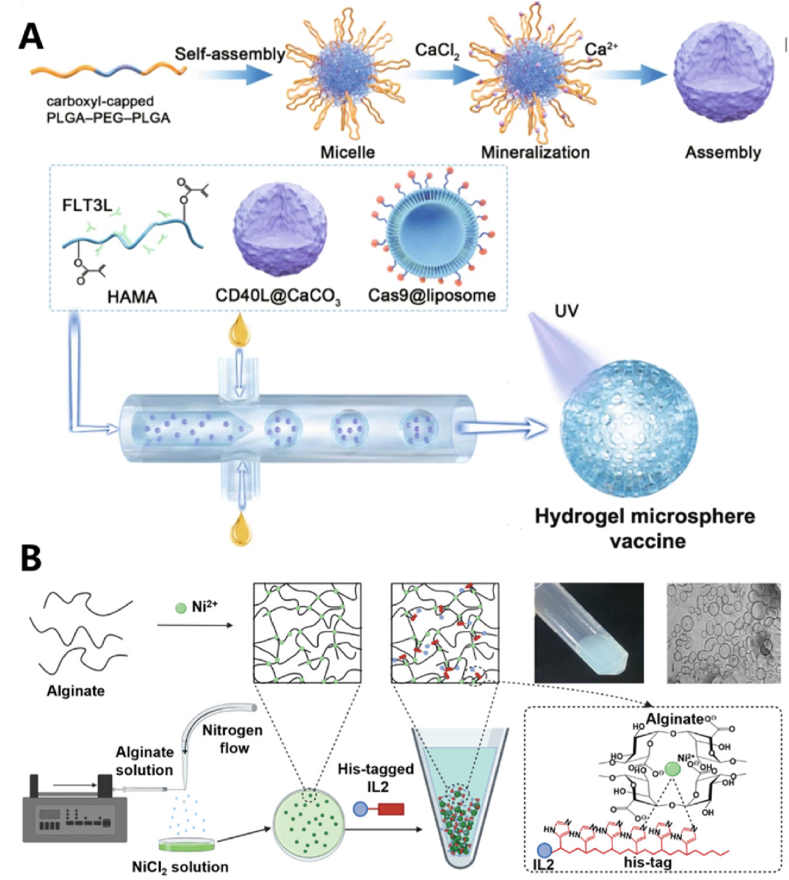
(A) Schematic illustration of the fabrication process for the interventional hydrogel microsphere vaccine, including Cas9@liposome nanoparticles. (B) The fabrication of Ni-ALGMSs and their specific encapsulation of His-labeled peptides or proteins. Reproduced with permission [52,105]. Copyright 2022, John Wiley and Sons. Copyright 2023, Springer Nature Limited.

Liu et al. encapsulated the Cas9 plasmid and CD40L individually within liposomes and CaCO_3_ nanoparticles. These were then combined with the FLT3L cytokine and hyaluronic acid (HA) to create a hydrogel microsphere vaccine (Fig. 11A) [105]. This formulation promotes CDC1-mediated antigen cross-presentation and triggers a cascade of CD8T cell activation, thereby transforming the immune-inert tumor microenvironment into an immune-active one. As a result, it significantly improves survival and inhibits the growth of distant metastases.

Xiong et al. reported the development of nickel alginate hydrogel microspheres (Ni-ALGMs) capable of effectively loading interleukin 2 (IL2) and providing sustained release following intratumoral administration [52]. In this design, histidine (His)-tagged IL2 is incorporated into Ni-ALGMs through a coordination bond between the His tag and Ni^2+^ (Fig. 11B). Upon injection of IL2-loaded Ni-ALGMs into the tumor, IL2 is gradually released over an extended period. The hydrogel microspheres significantly enhanced T lymphocyte infiltration into tumors and effectively inhibited tumor growth in tumor model therapies. Notably, when combined with the αPD-1 immune checkpoint inhibitor, the treatment led to complete tumor regression in 3 out of 6 mice in a colon cancer model, achieving a 50 % cure rate.

#### 4.3.4 Organoid construction

Hydrogel microspheres have been applied to treat various organ lesions in the human body, including those in the liver [106], eyes [93], and intestines [11], among others [7,107]. Chen et al. constructed a GelMA microgel encapsulating Lipo-Ebselen liposomes and polydopamine (PDA) [108]. The polyphenol groups in PDA were attached to the round window membrane (RWM) via high-strength coordination and hydrogen bonds, endowing the microspheres with the ability to adhere to the middle ear cavity. In a mouse model of noise-induced hearing loss (NIHL), the microspheres remained in the middle ear and gradually released drugs, leading to a significant improvement in hearing in the mice.

Ding et al. [109] developed a composite hydrogel microsphere material loaded with epidermal growth factor (EGF) for hemostasis and liver regeneration. The chitosan hydrogel microspheres were prepared using an emulsion crosslinking method, encapsulating EGF and embedding it in GelMA hydrogel. EGF played a crucial role in tissue remodeling by enhancing extracellular matrix synthesis and promoting cell proliferation. Additionally, GelMA hydrogel provides hemostatic and sealing effects. In a mouse liver injury model, the CM/GelMA composite material rapidly sealed the bleeding site, achieving hemostasis in approximately 10 s, which was significantly faster than GelMA hydrogel (34 ± 11 s) or gauze (89.92 ± 14.7 s). Furthermore, the CM/GelMA composite effectively prevented the rapid clearance of exogenous EGF, reducing the inflammatory response while maintaining a moist environment through its absorption properties. This facilitated hepatocyte proliferation, ultimately accelerating wound healing and liver regeneration.

### 4.4 Other applications

With continuous advancements in hydrogel microsphere research, these materials have demonstrated expanding applications beyond their initial uses. For example, peripheral nerve injury, a common type of nerve damage, is typically treated through surgical intervention or physical therapy. Javanmardi et al. developed hyaluronic acid microparticles loaded with dexamethasone (Dex) [110], where the high surface area of the microspheres facilitated rapid Dex release, effectively reducing neuroinflammation post-injury. In a rat model of sciatic neuropathy, proanthocyanidin (PA)-gelatin hydrogel coatings on these microspheres promoted sensory axon regeneration, significantly enhancing functional recovery.

Beyond these applications, hydrogel microspheres hold significant potential in gene therapy by serving as carriers for the controlled release of therapeutic nucleic acids and bioactive molecules, such as miRNA, plasmids, and exosomes. Their three-dimensional network structure effectively protects genetic materials from rapid degradation in vivo, while enabling sustained and regulated release to enhance gene delivery efficiency. Additionally, integrin-specific hydrogels (e.g., α3/α5β1 integrin-binding materials) can modulate cellular behavior by promoting adhesion between gene carriers and target cells, thereby improving delivery outcomes.

Moreover, hydrogel microspheres provide promising opportunities in **organoid repair**, particularly by enhancing vascularization and cellular organization within engineered tissues. Engineered hydrogel scaffolds can induce non-leaky angiogenesis through integrin-mediated interactions with the extracellular matrix, ensuring a stable nutrient supply essential for organoid growth and long-term maintenance [111]. By leveraging advances in biomaterials, biotechnology, and clinical medicine, hydrogel microspheres may further contribute to precision medicine strategies in regenerative medicine, ultimately facilitating the reconstruction of complex tissue injuries.

## 5 Conclusion

Hydrogel microspheres have emerged as a focal point in life science research due to their unique structures and versatile properties. Significant advancements have been made in material engineering, biofunctionalization, and biomedical applications, demonstrating their potential in diverse therapeutic and regenerative medicine fields. This review provides a comprehensive overview of recent progress, summarizing key strategies for material engineering strategies, biofunctionalization, and biomedical applications, while offering insights and reference strategies for future research and practical applications. However, despite these advances, challenges such as limited mechanical strength, scalability issues, and the need for enhanced biological adaptability still hinder their broader application. Future research should focus on overcoming these limitations by exploring innovative materials and fabrication techniques. For instance, the integration of double-network hydrogels, nanocomposites, and tissue-specific extracellular matrix components could improve mechanical robustness and cytocompatibility, making hydrogel microspheres more suitable for in vivo applications. Moreover, optimizing crosslinking strategies and surface modifications can enhance their structural integrity, degradation control, and functional responsiveness, further expanding their therapeutic potential. Additionally, the transition from laboratory-scale research to large-scale production remains a critical hurdle, requiring the incorporation of advanced biomanufacturing technologies such as 3D and 4D bioprinting to achieve precise control over microsphere size, spatial organization, and dynamic adaptability. Furthermore, emerging self-assembly strategies present a novel direction for hydrogel microsphere fabrication, allowing the development of self-healing and multifunctional microsphere-based hydrogels that could revolutionize minimally invasive therapies and personalized medicine [[112], [113], [114]]. As interdisciplinary collaboration continues to drive innovation in biomaterials and biomedical engineering, hydrogel microspheres are expected to evolve into more sophisticated, versatile, and clinically relevant biomaterials, providing new opportunities for precision medicine and regenerative therapies.

## CRediT authorship contribution statement

**Junjiang Yue:** Writing – review & editing, Writing – original draft. **Zhengbiao Liu:** Writing – original draft, Methodology, Investigation. **Lu Wang:** Methodology, Investigation. **Miao Wang:** Writing – review & editing, Investigation, Funding acquisition, Conceptualization. **Guoqing Pan:** Writing – review & editing, Validation, Supervision, Project administration, Investigation, Formal analysis, Conceptualization.

## Declaration of competing interest

The authors declare that they have no known competing financial interests or personal relationships that could have appeared to influence the work reported in this paper.

## Data Availability

No data was used for the research described in the article.
